# Astaxanthin Protection against Neuronal Excitotoxicity via Glutamate Receptor Inhibition and Improvement of Mitochondrial Function

**DOI:** 10.3390/md20100645

**Published:** 2022-10-18

**Authors:** Swapna Kannothum Kandy, Madhura Milind Nimonkar, Suravi Sasmita Dash, Bhupesh Mehta, Yogananda S. Markandeya

**Affiliations:** Department of Biophysics, National Institute of Mental Health and Neuroscience (NIMHANS), Bengaluru 560029, India

**Keywords:** astaxanthin, excitotoxicity, mitochondria, calcium, reactive oxygen species, cortical neurons

## Abstract

Excitotoxicity is known to associate with neurodegenerative diseases, such as Alzheimer’s disease, Parkinson’s disease, Amyotrophic lateral sclerosis and Huntington’s disease, as well as aging, stroke, trauma, ischemia and epilepsy. Excessive release of glutamate, overactivation of glutamate receptors, calcium overload, mitochondrial dysfunction and excessive reactive oxygen species (ROS) formation are a few of the suggested key mechanisms. Astaxanthin (AST), a carotenoid, is known to act as an antioxidant and protect neurons from excitotoxic injuries. However, the exact molecular mechanism of AST neuroprotection is not clear. Thus, in this study, we investigated the role of AST in neuroprotection in excitotoxicity. We utilized primary cortical neuronal culture and live cell fluorescence imaging for the study. Our results suggest that AST prevents neuronal death, reduces ROS formation and decreases the abnormal mitochondrial membrane depolarization induced by excitotoxic glutamate insult. Additionally, AST modulates intracellular calcium levels by inhibiting peak and irreversible secondary sustained calcium levels in neurons. Furthermore, AST regulates the ionotropic glutamate subtype receptors NMDA, AMPA, KA and mitochondrial calcium. Moreover, AST decreases NMDA and AMPA receptor protein expression levels, while KA remains unaffected. Overall, our results indicate that AST protects neurons from excitotoxic neuronal injury by regulating ionotropic glutamate receptors, cytosolic secondary calcium rise and mitochondrial calcium buffering. Hence, AST could be a promising therapeutic agent against excitotoxic insults in neurodegenerative diseases.

## 1. Introduction

Glutamate is a major excitatory amino acid neurotransmitter of the central nervous system, known to regulate nociception, motor function, synaptic plasticity, long-term potentiation, learning and memory [1,2]. Glutamate exerts its action by binding to the three ionotropic glutamate receptors, namely the N-methyl-D-aspartate (NMDA), amino-acid-3-hydroxy-5-methyl-isoxazol-4-propionic acid (AMPA) and Kainate (KA) receptors, as well as to the metabotropic glutamate receptors (mGluR) [3,4,5]. In addition to its physiological role, glutamate in excess results in neuronal excitotoxicity, observed in both in-vitro and in-vivo models [6,7]. Excitotoxicity has been suggested to be the final converging mechanism involved in several neurological disorders such as epilepsy, Parkinson’s disease (PD) and Amyotrophic lateral sclerosis (ALS), to name a few [8,9]. During excitotoxicity, overactivation of ligand-gated ionotropic glutamate receptors on the glutamatergic neurons leads to the excessive influx of Ca^2+^ from the extracellular space into the cell, thereby causing neuronal toxicity [10], as reported in the cortical, hippocampal, spinal, striatal and cerebral granular neuronal cultures [11,12,13,14,15]. The massive influx of calcium is known to trigger a cascade of activities, such as activation of calpain, mitochondrial dysfunction, increased generation of reactive oxygen species (ROS), activation of nitric oxide synthase, ER-stress and initiation of apoptotic signaling followed by cell death [16,17,18,19,20,21,22,23]. Furthermore, NMDARs tend to provoke a biphasic calcium response in cultured neurons: an initial rapid influx which is recovered to basal level and the other irreversible large secondary sustained calcium response (SSCR), commonly known as delayed calcium deregulation (DCD) correlated with neuronal death [11]. Several glutamate receptor antagonists, including memantine and perampanel, showed protection against excitotoxicity in animal model, but did not succeed in human trials [6]. Hence, there is a need to identify new pharmacological agents to protect neurons from excitotoxicity. Previous studies have reported neuroprotective effects of Astaxanthin (AST) in experimental models of cerebral ischemia, ischemic stroke and oxidative stress [24].

AST, an orange-red carotenoid, is largely found in aquatic organisms, available to some extent in Basidiomycetes, *Adonis amurensis*, *Haematococcus pluvialis* and members of Alphaproteobacteria [24,25,26]. AST is a fat-soluble compound that effectively crosses the blood–brain barrier [24,27]. AST is found to be safe for consumption by humans, with minimal mutagenicity, teratogenicity, embryotoxicity and reproductive toxicity [24,28]. It also maintains mitochondrial function, demonstrates higher antioxidant activity than vitamin E and is stronger than other carotenoids [24,27,29,30,31]. AST is also suggested to be an anti-inflammatory, antineoplastic, cardioprotective and neuroprotective agent [32,33,34,35]. Studies from PC-12, SH-SY5Y cells indicate that AST protects neurons from excitotoxic insult by inhibiting the intracellular calcium rise and ROS [36,37]. In a rat spinal cord injury model, AST improved motor functions with decreased cellular apoptosis [38]. In cortical neuronal cultures, AST decreased the mRNA levels of NMDA receptors subunits and showed an increase in the expression of voltage gated calcium channel subunit, A1D [39]. However, the mechanism of AST in regulating intracellular calcium overload, ROS formation and mitochondrial membrane potential (Ψm) in neuronal excitotoxicity is still not clear. 

In the present study, we investigated the protective role of AST on glutamate toxicity in cultured rat cortical neurons. Our results demonstrate that AST attenuates glutamate-induced neuronal cell death, as well as significantly reduces elevation in intracellular calcium concentration ([Ca^2+^]i) in cortical neurons upon glutamate exposure. It also attenuates the secondary sustained [Ca^2+^]i, response, mediated by glutamate subtype receptors such as NMDA, AMPA and KA. However, AST did not affect the voltage-dependent calcium channel response on KCl stimulation. Furthermore, AST significantly inhibited ROS formation and improved mitochondrial function. Overall, our data demonstrate that AST protects neurons from glutamate toxicity by reducing intracellular calcium overload and inhibiting mitochondrial calcium uptake.

## 2. Results

### 2.1. AST Modulates [Ca^2+^]i Dynamics in Excitotoxicity

**Figure 1:**
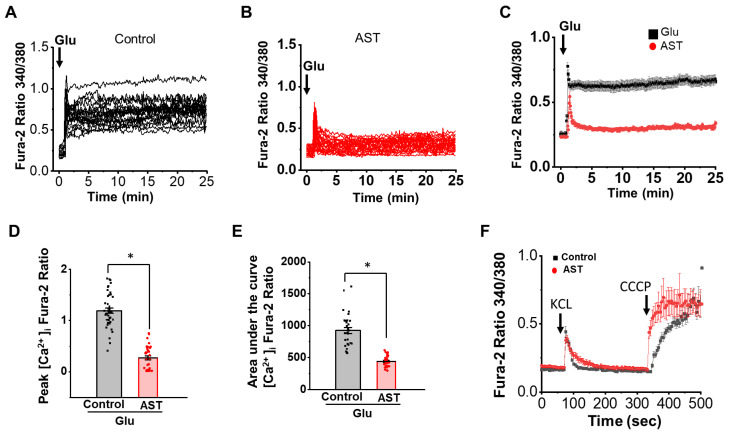
Astaxanthin preincubated cortical neurons exhibit reduced glutamate evoked [Ca^2+^]i increase. (**A**,**B**) Representative traces for Fura-2AM fluorescence indicating [Ca^2+^]i levels in neurons following glutamate (100 μM glutamate + 10 μM glycine) stimulation in control (*n* = 23) and AST preincubated neurons (*n* = 19). (**C**) Average intracellular calcium response of control and AST pretreated neurons to glutamate stimulation. (**D**,**E**) Dot plots for peak rise and area under the curve of [Ca^2+^]i response in control and AST preincubated neurons upon stimulation with glutamate. (**F**) Average [Ca^2+^]i response in control (*n* = 50) and AST (*n* = 46) preincubated neurons stimulated with 60 mM KCl followed by CCCP (10 μM) application. Arrow heads indicate point of application. Data represent mean ± SEM, * *p* < 0.05, from 3–5 experiments.

Glutamate is known to cause initial rapid Ca^2+^ influx and SSCR in several neuronal cultures, including cortical neurons. Earlier studies have suggested that glutamate toxicity in neurons is caused by the excessive influx of extracellular calcium and DCD [40]. Therefore, we investigated the effects of AST on glutamate-mediated intracellular calcium dynamics in 13–15 days in-vitro (DIV) cortical neurons obtained from postnatal day-one Wistar rat pups. Excitotoxicity was induced by treating neurons with 100 µM glutamate and 10 µM glycine for 25 min in magnesium free physiological buffer. We have used 100 µM glutamate +10 µM glycine, in all our experiments while using glutamate stimulation, unless stated otherwise. Intracellular calcium [Ca^2+^]i was measured using Fura-2AM dye. The basal calcium levels for both control and AST (50 µM, 48 h) pretreated neurons were comparable. Cortical neurons upon glutamate application displayed excessive increase in [Ca^2+^]i in a biphasic manner and a primary rapid calcium influx (0.8890 ± 0.06, *n* = 23) followed by a SSCR. The AST pretreated neurons, however, showed a significant reduction in both the primary Ca^2+^ influx (0.6304 ± 0.05, *n* = 19) as well as in the SSCR upon glutamate application (Figure 1A–E) and a faster recovery of [Ca^2+^]i to baseline in comparison to the non-AST pretreated controls. 

The reduced calcium influx in AST pretreated cultures may be a result of action of AST on the voltage gated calcium channels, well known for the rise in [Ca^2+^]i in neurons upon plasma membrane depolarization. Therefore, to examine whether AST affects non-glutamatergic or membrane depolarization induced Ca^2+^ influx, we depolarized cortical neurons using KCl (60 mM). Both AST treated and non-treated cortical neurons in cultures displayed a KCl (60 mM) evoked rapid increase in [Ca^2+^]i, (Control 0.440 ± 0.040, *n* = 50, AST 0.378 ± 0.022, *n* = 46) and with a similar pattern of recovery to baseline(Figure 1F). This ruled out the involvement of voltage activated calcium channels in glutamate-mediated calcium influx in AST pretreated cortical neurons. These results suggest that AST inhibits glutamate mediated excessive elevation in [Ca^2+^]i and SSCR.

### 2.2. AST Attenuates Excitotoxic Neuronal Death 

**Figure 2:**
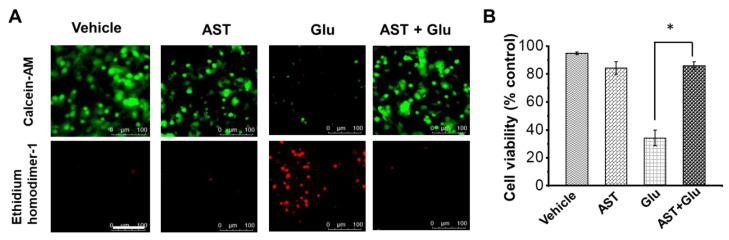
Astaxanthin increases cell survivability in cortical neurons from excitotoxicity. (**A**) Representative images showing live and dead neurons from control, glutamate, AST and AST with glutamate. Live neurons were labelled with Calcine-AM (green) and dead neurons were labelled with Ethidium homodimer-1 (red). (**B**) Bar graph represents viability of neurons to vehicle (95%), glutamate (34.2%), AST (87%) and AST with glutamate treatment (88%). Cell viability was measured 24 h after glutamate/vehicle treatment. Data are represented as mean ± SEM from N = 3 different experiments, with 10 separate fields captured from each coverslip (* *p* < 0.05).

To test out glutamate toxicity in cortical neurons and the attenuation of toxicity by AST, we preincubated primary cortical neurons with 50 µM AST for 48 h, followed by 25 min of glutamate stimulation. Then, 24 h post excitotoxic insult to the cortical neurons in cultures, we performed cell viability assay using the fluorescent dye Calcein-AM and EthD-1 [5,41]. AST pretreated cortical neurons in cultures showed significantly increased viability (53%, N = 3) upon glutamate exposure when compared to the AST non-treated cultures (34% N = 3) (Figure 2A). Cell viability in cortical neuron cultures pretreatment with AST alone (87%, N = 3) was comparable with the non-treated controls (95%, N = 3) (Figure 2B). These results suggest that AST pretreatment increases the cellular viability of cortical neurons upon glutamate excitotoxicity.

### 2.3. Chronic Treatment of AST Decreases NMDA, AMPA and KA Receptor-Mediated Ca^2+^ Influx and Their Protein Expression

**Figure 3:**
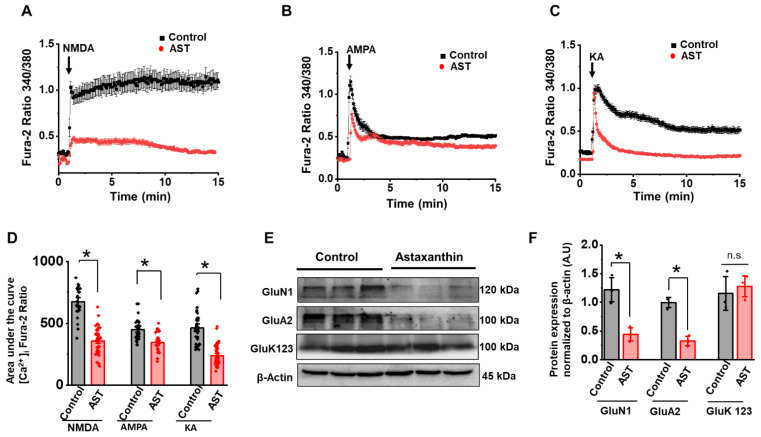
Astaxanthin inhibits a [Ca^2+^]i increase in cortical neurons upon ionotropic glutamate receptor activation. (**A**–**C**) The average [Ca^2+^]i response in control (black) and AST (red) preincubated neurons stimulated with 50 μM each of NMDA (+ 5 μM glycine), AMPA and KA for 15 min (NMDA: Con *n* = 23, AST *n* = 42; AMPA: con *n* = 27, AST *n* = 23; KA con *n* = 30, AST *n* = 40). (**D**) Dot plot representing the total calcium (area under the curve) after 15 min of NMDA, AMPA and KA stimulation. Arrow heads indicate point of glutamate receptor agonist applications. (**E**) Representative protein expression levels of NMDA (GluN1), AMPA (GluA2) and KA (GluK123) detected by the Western blot analysis with β-actin as the internal reference (individual Western blots figure are provided in Figure S1). (**F**) Dot plot indicate the average normalized protein expression for GluN1, GluA2 and GluK123. Data are represented as mean ± SEM from 3–4 different experiments, * *p* < 0.05. n.s: non-significant.

To identify the effect of AST on Ca^2+^ influx in cortical neurons upon ionotropic glutamate receptor stimulation, we exposed neurons in cultures for 25 min with the ionotropic glutamate receptor agonists, i.e., NMDA (50 µM with 5 µM glycine), AMPA (50 µM) and KA (50 µM), and imaged for 15 min after agonist application (Figure 3A–F). All cortical neurons displayed robust calcium influx upon exposure to the ionotropic glutamate receptor agonists. Of the three ionotropic receptor agonists, NMDA induced a biphasic [Ca^2+^]i response which was similar to the one observed upon glutamate application. Chronic AST-treated neurons (50 µM, 48 h) upon NMDA stimulation showed a 51% reduction in peak [Ca^2+^]i rise (NMDA 1.029 ± 0.06, *n* = 23; NMDA + AST 0.464 ± 0.06, *n* = 42) as well as a 51% decrease in the total calcium influx compared to that of NMDA alone (NMDA 713.11 ± 21.9, *n* = 23; NMDA + AST 349.79 ± 14.2, *n* = 42) (Figure 3A). Conversely, the AMPA receptor stimulation caused a monophasic [Ca^2+^]i response and chronic AST treated neurons showed inhibition of peak [Ca^2+^]i response (0.76 ± 0.04, *n* = 23) (Figure 3B). The total [Ca^2+^]i influx in chronic AST treated neurons was abridged by 20% with respect to AMPA alone (AMPA7 1.10 ± 0.07, *n* = 27; AMPA + AST 0.76 ± 0.04, *n* = 23). The total [Ca^2+^]i influx in chronic AST-treated neurons was abridged by 20% with respect to AMPA alone (AMPA 446.68 ± 14.3, *n* = 27; AMPA +AST 359.88 ± 17.5 *n* = 23). Additionally, the KA receptor stimulation caused a monophasic [Ca^2+^]i response with a delayed recovery to their baseline [Ca^2+^]i. In contrast, the chronic AST treated neurons showed inhibition of peak [Ca^2+^]i response (KA 0.985 ± 0.5, *n* = 30; KA + AST 0.99 ± 0.8, *n* = 40) and faster recovery to baseline [Ca^2+^]i upon KAR stimulation (Figure 3C). The total [Ca^2+^]i influx in chronic AST-treated neurons was abridged by 43% with respect to KA alone (KA 439.57 ± 19.19 *n* = 30; KA + AST 247.92 ± 13.43, *n* = 40) (Figure 3C). 

To understand the mechanism of AST inhibition on the glutamate receptors-mediated calcium influx, we next evaluated the NMDA, AMPA and KA receptor protein expression levels. Neurons were preincubated with 50 μM AST for 48 h and cell lysates were analyzed using Western blot by probing with their respective antibodies. As shown in Figure 3E, AST preincubation significantly reduced the protein expression of NMDA (GluN1) and AMPA (GluA2), whereas no change in the protein expression KA (GluK123) was observed. The densitometry analysis of the western blots showed a 63% and 67% reduction in the expression of GluN1 and GluA2, respectively (Figure 3F). Overall, our data demonstrate that chronic AST treatment reduces the calcium influx through NMDA, AMPA and KA receptor, well supported by the reduced expression of GluN1 and GluA2.

### 2.4. Acute Treatment of AST Inhibits NMDA, AMPA and KA Receptor [Ca^2+^] Response 

**Figure 4:**
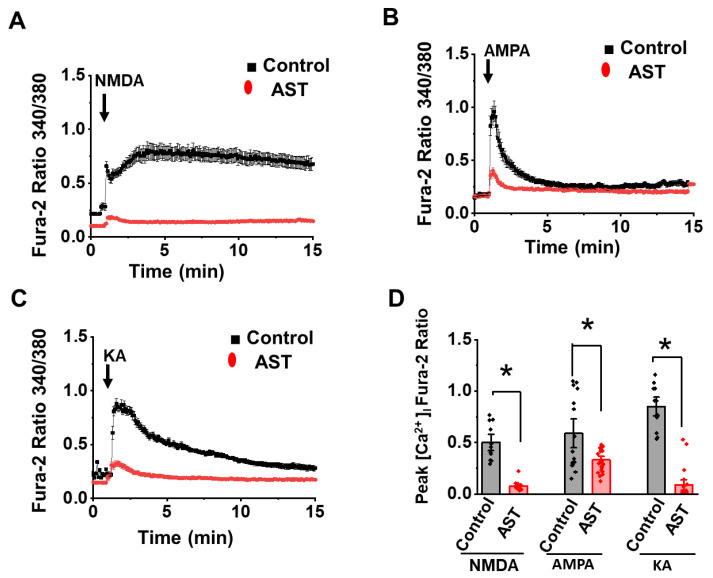
Acute treatment of Astaxanthin inhibits the [Ca^2+]^i increase in cortical neurons upon ionotropic glutamate receptor activation. (**A**–**C**) Average [Ca^2+^]i response in control (black) and 5 min of AST-treated neurons (red) stimulated with NMDA (50 μM + 5 μM glycine), AMPA (50 μM) and KA (50 μM). (**D**) Dot plot representing the peak calcium rise after 15 min of agonist (arrow head) application. Data are represented as mean ± SEM from three different experiments (*n* > 23), * *p* < 0.05.

To evaluate whether AST directly interacts with the ionotropic glutamate receptors, cortical neurons were stimulated with NMDA (50 µM with 5 µM glycine), AMPA (50 µM) and KA (50 µM) in presence of AST (50 µM). As shown in Figure 4A–D, neurons showed robust increase in intracellular calcium upon NMDA, AMPA and KA application. However, acute treatment (5 min) of AST reduced the peak calcium. In presence of AST, NMDA stimulation showed an 80% inhibition of intracellular calcium rise (NMDA control 0.518 ± 0.04, *n* = 18; NMDA + AST 0.101 ± 0.01, *n* = 22). In the presence of AST, a 51% reduction in peak calcium was observed upon AMPA application (AMPA control 0.655 ± 0.07, *n* = 22; AMPA + AST 306 ± 0.02, *n* = 30). AST reduced the calcium response in neurons by 89% upon KA application (KA control 0.837 ± 0.05, *n* = 19; KA + AST 0.093 ± 0.02, *n* = 28). Thus, our results suggest that AST directly inhibits the ionotropic glutamate receptor response at the plasma membrane level.

### 2.5. AST Abrogates Glutamate-Mediated Increase in Mitochondrial Membrane Potential (Ψm)

**Figure 5:**
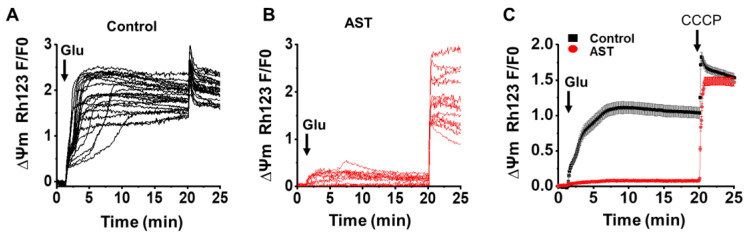
Astaxanthin inhibits glutamate-mediated alteration in mitochondrial membrane potential (Ψm) in cortical neurons. (**A**,**B**) The representative traces of Rh123 fluorescence indicating ∆Ψm in neurons following glutamate stimulation in control and AST preincubated neurons. (**C**) Average ∆Ψm response in control (*n* = 36) and AST (*n* = 35) preincubated neurons stimulated with glutamate; CCCP (1 μM) was added to see the complete depolarization of mitochondria. Arrow heads indicate the point of application. Data are represented as mean ± SEM from 4 different experiments.

To check the effect of AST on the ∆Ψm upon glutamate receptor stimulation, we used the fluorescent Ψm dye Rh123 (5 µM). As shown in the Figure 5 glutamate application to the cortical neurons caused a large surge in Rh123 fluorescence (1.92 ± 0.07, *n* = 36), whereas a significant reduction,(86.9%, *p* < 0.05) in the Rh123 fluorescence was observed upon glutamate stimulation in the AST pretreated cultures (0.25 ± 0.01, *n* = 35). In each of our Rh123 fluorescence recording experiments, we uncoupled the mitochondrial membrane potential in the last steps of recordings by using the mitochondrial protonophore, CCCP (1 µM), thereby giving us an idea about the overall extent of mitochondrial depolarization due to calcium accumulation in the mitochondria upon glutamate application. Figure 5C shows the change in Rh123 florescence intensity observed upon the addition of CCCP (1 µM) in cortical neurons. In comparison to the controls (0.8 ± 0.04, *n* = 36), AST pretreated neurons upon CCCP addition showed an intense increase in Rh123 fluorescence (2.03 ± 0.1, *n* = 35). These results indicate that AST improves the mitochondrial function during excitotoxic conditions. 

### 2.6. AST Inhibits Glutamate-Induced Reactive Oxygen Species (ROS)

**Figure 6:**
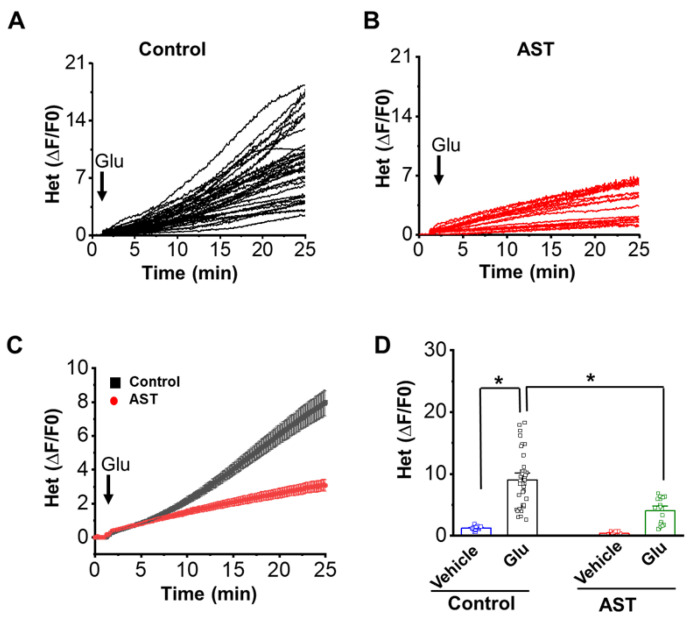
Astaxanthin inhibits glutamate induced ROS formation in cortical neurons. (**A**,**B**) The representative traces of change in ROS formation upon glutamate (for 25 min) stimulation recorded as an increase in HEt fluorescence in control (*n* = 37) and AST preincubated (*n* = 23) cortical neurons. (**C**) Average ROS formation upon glutamate stimulation in vehicle and AST preincubated neurons. (**D**) Dot plot represents the mean ROS formation in control and AST pretreated neurons upon vehicle/glutamate stimulation. Arrow heads indicate point of glutamate applications. Data represent mean ± SEM, * *p* < 0.05, from 5 experiments.

To determine the impact of AST on glutamate-mediated ROS formation, neurons were stimulated with glutamate in presence of dihydroethidium (5 µM), a fluorescent ROS dye. Figure 6 shows glutamate exposure producing a two-fold increase in ROS (9.083 ± 0.7, *n* = 37) formation in cortical neurons. However, AST preincubated neurons (4.075 ± 0.48, *n* = 37) showed a diminished (55%) ROS formation upon glutamate stimulation. The ROS levels without glutamate application in the control neurons in cultures was 1.2331 ± 0.05, *n* = 24 and in AST pretreated cultures was 0.4027 ± 0.04, *n* = 23. These results suggest that AST inhibits glutamate-mediated increase in ROS formation in the cortical neurons.

### 2.7. AST Affects Mitochondrial Calcium

**Figure 7:**
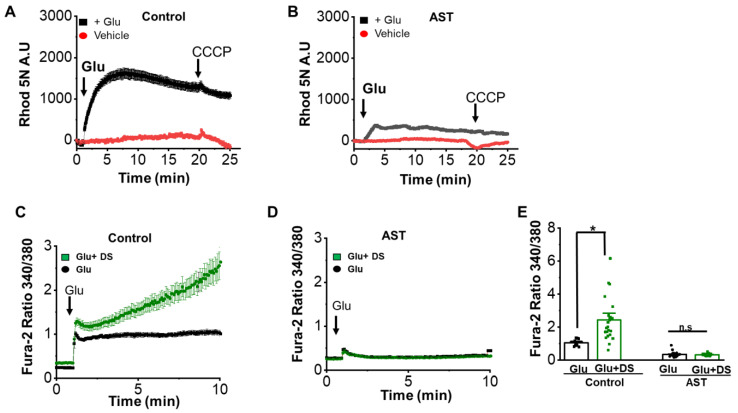
Astaxanthin inhibits mitochondrial calcium uptake on glutamate stimulation. (**A**,**B**) Average Rhod 5N fluorescence indicating [Ca^2+^]m in neurons following glutamate stimulation in control (*n* = 33) and AST preincubated neurons (*n* = 49). (**C**,**D**) Average [Ca^2+^]i in neurons following glutamate stimulation in control (*n* = 14) and AST (*n* = 23) preincubated neurons in presence of mitochondrial Ca^2+^ uniporter complex blocker 30 μM DS16570511 (DS). (**E**) Dot plot representing mean [Ca^2+^]i after 10 min of glutamate addition. Data are represented as mean ± SEM from 4 different experiments (* *p* < 0.05). n.s: non-significant.

To examine the effect of AST on mitochondrial calcium ([Ca^2+^]m) accumulation. We measured the [Ca^2+^]m accumulation upon glutamate application, by using a low affinity mitochondrial calcium sensitive dye, Rhod 5N. As shown in Figure 7A,B, in comparison to the control cultures (1550.4 ± 2.4, *n* = 36), AST pretreated cultures (234 ± 2.3, *n* = 49) showed a significant decrease in the Rhod 5N fluorescence upon glutamate application. The decrease could be the result of lower buffering of calcium by the mitochondria. 

It is known that the mitochondria buffers excess of cytosolic calcium through the mitochondrial calcium uniporters (MCU) [42]. To test the functioning of the MCU upon glutamate exposure in our culture conditions, we used DS 16570511(DS), a potent mitochondrial uniporter blocker [42]. In the control conditions without AST preincubation, glutamate exposure caused a huge rise in [Ca^2+^]i in the presence of DS (2.42 ± 0.2, *n* = 23), when compared to the sole application of glutamate(1.04 ± 0.05, *n* = 30) (Figure 7C,E). Interestingly, AST pretreated neurons showed no significant difference in the cytosolic calcium rise upon glutamate stimulation in both the presence (0.324 ± 0.01, *n* = 43) or absence of DS (0.3502 ± 0.17, *n* = 30) (Figure 7D,E), thereby indicating that the mitochondrial Ca^2+^ accumulation is unaffected upon AST pretreatment. The above results suggest that AST inhibits mitochondrial calcium uptake, without affecting MCU function.

## 3. Discussion

Emerging literature indicates that Astaxanthin, a natural antioxidant, works as a promising therapeutic agent in both in vitro and in vivo models of Alzheimer’s disease, Parkinson’s disease, epilepsy, neuropathic pain and in various other models of neurodegenerative disorders [40,43,44,45]. Earlier studies have shown that AST protects PC12 and SH-SY5Y cells against excitotoxicity, by inhibiting elevation in intracellular calcium levels and reactive oxygen species. However, the exact molecular mechanism is not clear. Hence, in this study, we investigated the effects of AST on intracellular calcium dynamics, mitochondrial function and ROS formation during excitotoxic condition in primary cortical neurons. Our results demonstrate that chronic treatment of AST to cortical neurons (1) alleviates the primary and irreversible secondary sustained [Ca^2+^]i response, (2) regulates the permeability of NMDA, AMPA, and KA receptors, (3) reduces the protein expression of NMDA and AMPA receptors, (4) inhibits mitochondrial calcium accumulation, (5) inhibits the abnormal ∆Ψm, ROS formation, and (6) promote neuronal survival. Furthermore, acute treatment of AST could directly inhibit glutamate subtype receptor calcium influx. 

It is firmly established that excess glutamate induces loss of neuronal survivability in primary neuronal cultures [6,18]. Correspondingly, we also found significant loss in neuronal survivability on glutamate addition. Interestingly, similar to the earlier observations [36,46], we observed high neuronal survivability upon glutamate exposure in AST preincubated cultures. In neurons, the glutamate toxicity is majorly due to the excessive intracellular calcium overload, which involves a rapid primary calcium influx and an irreversible SSCR [18,47]. The latter is suggested to be more toxic to the neurons [6]. The initial rapid primary response is through the activation of ligand gated ionotropic glutamate receptor, the NMDA, AMPA and KA receptors, and further due to the activation of the voltage-gated calcium channels. The SSCR is associate with the loss of mitochondrial function, which in turn depends on the primary influx of calcium into the neuron from the extracellular space [6]. Earlier reports by fluorometric analysis have suggested that AST attenuates the [Ca^2+^]i response in PC12 and SH-SY5Y cells upon glutamate application [36,46], and in hippocampal neurons upon NMDA application [27,37]. However, these studies have not demonstrated AST-mediated inhibition of both the primary Ca^2+^ influx as well as the SSCR upon ionotropic glutamate receptor agonists activation. Nevertheless, these studies have not shown AST inhibition of the AMPA and the KA receptors. Our result demonstrates that the AST-mediated inhibition of the calcium influx via NMDA and AMPA receptor activation can be due to a decrease in the protein expression levels of NMDA (GluN1) and AMPA (GluA2). This is in agreement with a recent report, where AST pretreatment resulted in the loss of NMDA receptor subunits mRNA expression in cultured primary cortical neurons [39]. In contrast to the NMDAR and AMPAR activation, AST did not affect the primary calcium response upon KA stimulation. It also did not alter the expression of KA (GluK123) receptor at the protein level. The fast recovery of the KA response may be an additional effect of AST on KA receptor gating property. Earlier literature shows that AST prevents neuronal loss, increases neuronal survivability and reduces seizure activity in kainic acid-induced seizures in rats [48]. Interestingly, chronic AST treatment demonstrates that AST causing changes in [Ca^2+^]i was in the order NMDA > KA > AMPA and partially attenuates receptor function. Results from the acute AST treatment suggest that AST could directly regulate NMDA, AMPA and KA receptors. This is in agreement with the recent in silico molecular docking study indicating that AST fits into NR2B binding pocket of NMDA receptor, causing an inhibitory effect [34]. 

The SSCR in excitotoxicity is known to be responsible for neuronal injury in cerebellar granular neurons and spinal neurons [11,13]. In our experiments, preincubation of neurons with AST inhibited SSCR in cultured cortical neurons, thus increasing the neuronal survivability. Several studies have demonstrated that SSCR results due to the release of [Ca^2+^]m into the cytosol, which is accumulated during initial peak after glutamate stimulation. This Ca^2+^ release from the mitochondria is due to the loss of Ψm [49,50]. Similar to the earlier reports, we also observed loss of Ψm upon glutamate stimulation, which was not observed in AST pretreated cultures, and thereby, increased neuronal survivability. 

Previous studies have suggested that glutamate receptor over activation results in intra cellular calcium overload, followed by increased production of ROS [50,51,52]. The Ca^2+^-dependent ROS formation is suggested to be through the activation of phospholipase A2, xanthin oxidase, nitric oxide synthase and mitochondria [53,54]. We measured ROS using HEt, a superoxide radical sensitive dye, indicating that the ROS generation has a mitochondrial origin [55,56]. Earlier studies have reported that the rapid sequestration of intracellular calcium through the mitochondrial calcium uniporter (MCU) is majorly responsible for the ROS formation in neurons during excitotoxicity [18]. Due to the accumulation of excessive cations in the mitochondria, dissipation of the Ψm occurs, followed by the opening of MPTP, which causes leakage of cytochrome-c (Cyt-c), followed by a cascade of events leading to neuronal apoptosis and death [57]. Consistent with the earlier reports, we also observed an increase in ROS formation in glutamate-treated cortical neurons, with a two-fold decrease in ROS formation upon AST pretreatment, an observation in line with the decrease in [Ca^2+^]i and increase in neuronal survivability. This result corroborates with the report of AST inhibition of mitochondrial H_2_O_2_ upon Aβ toxicity in hippocampal neurons [27], as well as with the suppressing ROS formation in the rat model of transient cerebral ischemia [58]. Additionally, reports have suggested that AST inhibits the glutamate-mediated increase in ROS in SH-SY5Y and PC12 cells [36,46]. Interestingly, our results demonstrate that AST could inhibit both Ψm and [Ca^2+^]m accumulation during excitotoxicity, which were not performed by others. If the cytosolic Ca^2+^ concentration is below a threshold, the catastrophic event leading to excitotoxicity could be prevented. Furthermore, [Ca^2+^]m measurement with Rhod 5N showed a significant decrease in [Ca^2+^]m uptake in AST pretreated neurons compared to control cultures. In addition, mitochondrial uniporter blocker experiments showed no difference in intracellular calcium concentration of both AST-treated and -untreated neurons, indicating that AST limits [Ca^2+^]m uptake but not through the MCU. 

In conclusion, we believe that AST regulates the influx of calcium through glutamate receptors at the plasma membrane, and improves mitochondrial function by reducing calcium uptake, thereby protecting the neurons from excitotoxicity. 

## 4. Materials and Methods

### 4.1. Materials

Tissue culture chemicals were sourced from Invitrogen. Fluorescence-sensitive probes Fura-2AM, Rhodamine 123 (Rh123), Rhod 5N, Hydroethidine (HEt), Calcein acetoxymethyl ester (Calcein-AM) and Ethidium homodimer were from Invitrogen Thermo Fisher Scientific USA (Waltham, MA, USA). DS16570511 is from Cayman Chemical (Ann Arbor, MI, USA). AMPA is from Tocris. Astaxanthin, NMDA, KA, CCCP, Astaxanthin (≥97% HPLC) and all other chemicals were of analytical grade purity from Sigma-Aldrich (Merck, St Louis, MO, USA).

### 4.2. Experimental Animal

All the experiments were performed in accordance with the guidelines established by the Institutional Animal Care and Use Committee NIMHANS. Post-natal day-1 Wistar rat pups were obtained from the Central Animal Research Facility (CARF) of NIMHANS, Bengaluru, India. Care was taken to minimize discomfort to animals during all the procedures (IAEC Ref No: AEC/70/454/BP).

### 4.3. Preparation of Cortical Neuronal Culture

Cortical neuronal cultures were prepared from post-natal day-1 Wistar rat pups. After decapitation, the whole brain was removed and placed in ice cold Hanks’ balanced salt solution (HBSS), containing (in mM) 135 NaCl, 5.3 KCl, 4.0 NaHCO_3_, 5 KH_2_PO_4_, 3 Na_2_HPO_4_, 1.2 CaCl_2_, 1.2 MgCl_2_ and 10 Glucose. The cortical tissues were incubated in Ca^2+^ and Mg^2+^-free HBSS containing 0.25% trypsin for 20 min at 37 °C, after which trypsin was washed by rinsing tissue with Ca^2+^ and Mg^2+^ containing HBSS, followed a wash with Dulbecco’s Modified Eagle’s Medium (DMEM), supplemented with 10% horse serum and 10% fetal bovine serum. The tissues were triturated and plated on 16 mm poly-D-lysine-coated glass cover slips placed on a 35 mm plastic petri dish in DMEM supplemented with 10% horse serum and 10% fetal bovine serum (FBS). The cultures were maintained in 95% air and 5% CO_2_, at 37 °C in humidified atmosphere. On the third day, the cultures were treated with the mitotic inhibitor 10 µM cytosine arabinoside (AraC) to prevent proliferation of non-neuronal cells. On the fourth day, AraC was removed with fresh DMEM medium supplemented with 5% horse serum and 5% fetal bovine serum. Subsequently, media change was done on every third day until the 13–15th day in vitro (DIV). In all our experiments while stimulating with 100 µM glutamate, we have used +10 µM glycine, unless stated otherwise. While stimulating with NMDA, we have used 50 µM NMDA and 5 µM glycine as co-agonists in our experiments. 

### 4.4. Measurement of Intracellular Calcium

Intracellular calcium imaging was performed using high affinity fluorescent calcium indicator Fura-2AM. Neurons on the coverslips were loaded with Fura-2AM (5 µM) in normal physiological buffer (in mM): 125 NaCl, 5 KCl, 1.2 MgSO_4_, 1.8 CaCl_2_, 1.2 KH_2_PO_4_, 23 NaHCO_3_, 3 HEPES and 10 glucose, containing 0.01% Pluronic acid for 30 min at 37 °C. The dye-loaded coverslips were rinsed twice with fresh HEPES buffer and mounted on a perfusion chamber onto the stage of an inverted fluorescence microscope (Leica, Wetzlar, Germany). Fura-2AM loaded cells were alternately excited at 340 and 380 nm with a compact LED light source (CoolLED, Andover, UK), and images acquired every 5 s with a 12-bit Peltier cooled EM CCD camera (Andor Oxford Instruments, Belfast, UK), using an Apo 40X objective and appropriate filter. The acquired data were analyzed using LAS X Leica software (Leica, Wetzlar, Germany).

### 4.5. Measurement of Mitochondrial Membrane Potential (Ψm) and Mitochondrial Calcium ([Ca^2+^]m) 

Mitochondrial membrane potential was examined using rhodamine123 (Rh123), a cationic, amphipathic and relatively non-toxic dye that selectively stains mitochondria in proportion to their highly negative transmembrane potential. Rh123 accumulates in polarized mitochondria, which quenches the fluorescence signal, in response to mitochondrial depolarization fluorescence signal de-quenches and Rh123 signal increase [40]. Neurons were incubated with 5 µM Rh123 for 20 min at 37 °C. Rh123 signal was recorded by measuring changes in the emitted fluorescence intensity at 537 (Barrier filter BA515) with excitation at 490 nm. All Rh123 responses were normalized to the resting fluorescence, which was expressed as 100% to demonstrate the extent of stimulant-induced depolarization. Mitochondrial calcium was measured by incubating neurons with 5 µM of Rhod 5AM dye for 10 min and desertification for 30 min in the HBSS buffer [59]. The increase in mitochondrial calcium was monitored as the increase in the emitted fluorescence at 576 nm by exciting with a light of 552 nm using the barrier filter BA590.

### 4.6. Measurement of the Reactive Oxygen Species (ROS)

ROS generation was estimated by using Dihydroethidium (DHE). The super oxide radical generated will oxidize the DHE to ethidium (Het), a red fluorescent compound that chelates to DNA. DHE shows a blue fluorescence (absorption/emission: 355/420 nm) in cell cytoplasm until oxidization to form ethidium which becomes red fluorescent (absorption/emission: 470/590 nm). Coverslips containing neurons were mounted on perfusion chamber placed on inverted epifluorescence microscope. The area of interest was selected using phase contrast images. Perfused with normal buffer for 5 min, neurons were further treated with 5 µM HEt along with agonists. The ROS formation was monitored by measuring an increase in DHE fluorescence upon excitation at 520 nm and the emission at 635 nm through barrier filter BA590.

### 4.7. Neuronal Survival 

Neuronal viability was determined with Calcein acetoxymethyl ester (Calcein-AM) and EthD-1 staining. Calcein-AM is a non-fluorescent lipophilic ester that easily penetrates the cellular membrane. It is then rapidly cleaved by nonspecific cytosolic esterase to the fluorescent Calcein-AM [27]. Cells were rinsed twice with sterile HBSS, after a 5-min preincubation with HBSS alone; neurons were exposed to the appropriate agonists for 20 min. After stimulus exposure, neurons were incubated in CO_2_ incubator in culture medium for 24 h. Cells were then washed with fresh HBSS and incubated in 0.2 µM Calcein AM and 0.4 µM EthD-1 (Thermo Fisher Scientific Inc, Waltham, MA, USA) for 5 min, at room temperature. The images were collected at excitation/emission wave length of 485/530 and 520/610 nm, respectively, using a fluorescence-inverted microscope. Only viable neurons are able to cleave and trap the fluorescent Calcein product. For quantitation of viable neurons, cells were counted in 10 separate fields in each coverslip. The survival rate was expressed as the percentage ratio of average number of neurons in treated cultures and that of the parallel control culture.

### 4.8. Western Blot Analysis 

Cell lysates were made from 13–15 DIV control and AST treated cortical neurons, separated by SDS-PAGE and transferred to PVDF membrane. Nonspecific binding sites were blocked by 5% (*w*/*v*) dried skim milk in PBST (0.1% Tween 20). Membranes were then probed with specific primary antibodies GluN1 (NMDAR1, 1:1000, Alomone Lab Jerusalem, Israel), GluR2 (GluA2, 1:1000, Alomone Lab), GluK 123 (GluR 567, 1:2000, Santa Cruz, Dallas, TX, USA) and β-Actin, 1:2000, BD). Secondary antibody was either goat anti-mouse Ig (1:10,000, Sigma, Merck, St Louis, MO, USA) or goat anti-rabbit horseradish peroxidase (1:10,000, Sigma, Merck, St Louis, MO, USA) antibody. Blots were visualized using the peroxidase-based chemiluminescent detection kit by ECL, Femto LUCENT luminol solution (G-Biosciences, Louis, MO, USA).

### 4.9. Data Analysis

The data were analyzed using statistical software OriginPro 2019b (OriginLab Corporation, Northampton, MA, USA). The results are represented as mean ± SEM, observed difference was evaluated using unpaired two sample student’s *t* test. *p* ≤ 0.05 was considered statistically significant.

## Data Availability

Not applicable.

## Supplementary Materials

The following supporting information can be downloaded at: https://www.mdpi.com/article/10.3390/md20100645/s1, Figure S1: Western Blot.
