# Supplementary material for: Astaxanthin Protection against Neuronal Excitotoxicity via Glutamate Receptor Inhibition and Improvement of Mitochondrial Function
Source: Mar Drugs. 2022 Oct 18;20(10):645. doi: 10.3390/md20100645 (PMC9605357; doi:10.3390/md20100645)

Supplemental Data:

Astaxanthin Protection Against Neuronal Excitotoxicity is via Glutamate Receptor Inhibition and Improvement of Mitochondrial Function

Swapna Kannothum Kandy [1], Madhura Milind Nimonkar [1], Suravi Sasmita Dash[1], Bhupesh Mehta[1] and Yogananda S. Markandeya [1,*]

Western blot:

Cell lysates were made from 13-15 DIV Control and AST treated cortical neurons, separated by SDS-PAGE and transferred to polyvinylidene difluoride membranes. Nonspecific binding sites were blocked by 5% (w/v) dried skim milk in PBST (0.1% Tween 20). Membranes then probed with specific primary antibodies GluN1(NMDAR1)-1:1000, Alomone; GluR2(GluA2)-1:1000, Alomone; GluK 123(GluR 567)-1:2000, Santa Cruz; β-Actin- 1:2000; BD) followed by PBST washes (four times for 10 min each). After incubating with either goat anti mouse Ig (1:10,000, Sigma) or goat anti-rabbit IgG conjugated to horseradish peroxidase (1:10,000, Sigma) for 1 h, the membranes were washed (six times for 10min) with PBS detergent. Immunoreactivity was visualized using peroxidase-based chemiluminescent detection by ECL,Femto LUCENT luminol solution (G Bio Science) Gluk123 were striped and re probed in the GluN1 blot, hence beta-actin was same for both the Gluk123 and GluN1 blots

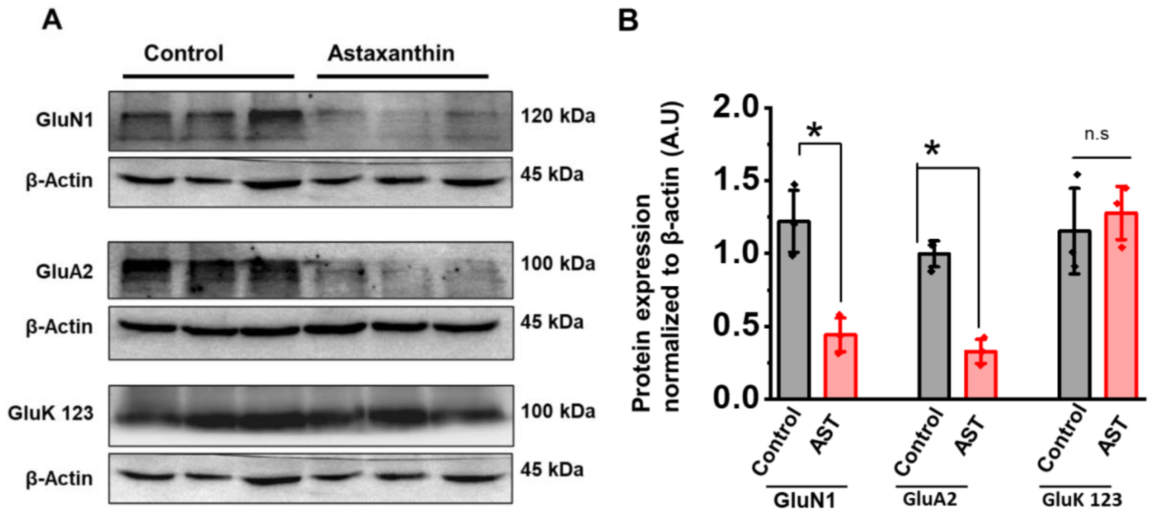

Supplement: Supplementary file 1 [file marinedrugs-20-00645-s001.zip › marinedrugs-1916631-supplementary.pdf]
